# The Physiologic Mechanisms of Paced QRS Narrowing During Left Bundle Branch Pacing in Right Bundle Branch Block Patients

**DOI:** 10.3389/fcvm.2022.835493

**Published:** 2022-03-09

**Authors:** Kailun Zhu, Yali Sun, Manxin Lin, Yingjian Deng, Linlin Li, Guiyang Li, Jianghai Liu, Xingcai Wan, Dong Chang, Qiang Li

**Affiliations:** ^1^Department of Cardiology, Xiamen Cardiovascular Hospital of Xiamen University, School of Medicine, Xiamen University, Xiamen, China; ^2^School of Medicine, Xiamen University, Xiamen, China

**Keywords:** left bundle branch pacing, right bundle branch block, QRS complex, longitudinal dissociation, anodal stimulation

## Abstract

Left bundle branch pacing (LBBP) is a physiological pacing technique that captures the left bundle branch (LBB) directly, causing the left ventricle (LV) to be excited earlier than the right ventricle (RV), resulting in a “iatrogenic” right bundle branch block (RBBB) pacing pattern. Several studies have recently shown that permanent LBBP can completely or partially narrow the wide QRS duration of the intrinsic RBBB in most patients with bradycardia, although the mechanisms by which this occurs has not been thoroughly investigated. This article presents a review of the LBBP in patients with intrinsic RBBB mentioned in current case reports and clinical studies, discussing the technique, possible mechanisms, future clinical explorations, and the feasibility of eliminating the interventricular dyssynchronization accompanied with LBBP.

## Introduction

For decades, right ventricular pacing (RVP) has been the standard treatment for patients with symptomatic bradyarrhythmia caused by sinus node dysfunction or atrioventricular conduction disease. RVP, however, has been established to cause electrical and mechanical dyssynchronization, which increases the risk of cardiac dysfunction, heart failure hospitalization, atrial fibrillation, and a higher mortality rate (1–3). Therefore, His bundle pacing (HBP), a physiologic pacing strategy, has been developed. Multiple observational studies have demonstrated the feasibility and therapeutic advantages of HBP in maintaining cardiac synchrony (4–9). However, the clinical applicability of HBP is restricted due to its high pacing threshold, low sensing amplitude, and technically challenging of implantation (6, 10, 11).

Left bundle branch pacing (LBBP) is a novel physiological pacing technique in which a Select Secure lead (Model 3830 69 cm, Medtronic Inc., Minneapolis, MN, United States) delivered through the Select Site preshaped sheath (C315HIS, Medtronic Inc., Minneapolis, MN, United States) is deeply rotated via a transventricular septal approach to capture the left bundle branch (LBB), including the trunk and its proximal branches. When compared to HBP, LBBP has been shown to be effective, feasible, and safe for correcting LBB block and maintaining physiological left ventricle (LV) activation, with a greater success rate and more stable lead parameters (12–16). But little is known about LBBP in patients with intrinsic right bundle branch block (RBBB) who have pacemaker indications.

## Left Bundle Branch Pacing Technique and ECG Features

The presence of a paced RBBB pattern is a required but insufficient criterion for confirmation of LBB capture with a sensitivity of 100% (17). When the lead is advancing from the right ventricular septum to the left, the morphology of paced QRS complex changes dynamically, as seen by the W-shaped notch at the nadir of the QRS complex in lead V1 gradually moving to the end of that and eventually presenting a pseudo-RBBB pattern (13, 14). This is because LBBP can directly capture the LBB, causing the excitation of the left ventricular lateral wall to be to accelerated while the excitation of the right ventricle (RV) to be delayed (17–20). LBBP can be divided into two types: selective LBBP (SLBBP), which involves only LBB capture, and non-selective LBBP (NSLBBP), which involves LBB and surrounding myocardium capture. At a low output, SLBBP is achieved, with a typical paced RBBB morphology (QRS duration > 0.12 s, rSR’ pattern in leads V1 and V2, wide and slurred S wave in leads V6 and I). At a high pacing output, NSLBBP is achieved, with an atypical paced RBBB morphology (QR pattern and narrow R without a distinct notch in lead V1, narrow and small S wave without a notch in leads V6 and I) (17, 21).

## Left Bundle Branch Pacing in Right Bundle Branch Block Patients

**FIGURE 1:**
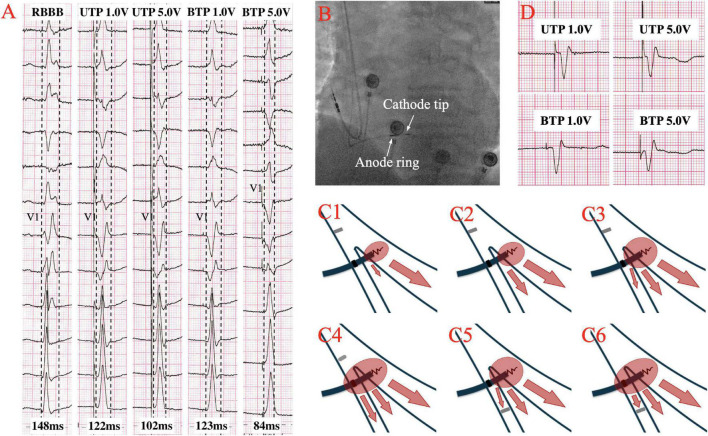
**(A)** The QRS morphologies of intrinsic right bundle branch block (RBBB) under different pacing configurations of left bundle branch pacing (LBBP) at speed of 50 mm/s. QRS duration is shortened from intrinsic 148–122 ms at low output unipolar LBBP, and is further shortened to 102 ms at high output. The paced QRS morphology does not change significantly in unipolar pacing with low or high outputs, both presented a Qr pattern in lead V1. At low output bipolar LBBP, the paced QRS duration is 123 ms, which was similar to the performance at low output unipolar LBBP. However, at high output bipolar LBBP, the paced QRS duration is shortened to 84ms, and the paced QRS morphology changes significantly, showing that the r’ wave at the end of the QRS in lead V1 disappeared, suggesting the RV delay was compensated. **(B)** Sheath angiography of 3830 lead after LBBP completion. **(C1–C6)** Schematic diagram of different pacing modes of LBBP performed on RBBB. **(C1–C3)** Low output unipolar LBBP only captures the left bundle branch (LBB) and a small portion of the surrounding myocardium to partially shorten the RBBB. High-output unipolar LBBP captures LBB and more surrounding myocardium, further shortening RBBB, and may even overcome the resistivity of fibrous sheaths of longitudinal dissociation to completely correct RBBB. **(C4)** High output bipolar LBBP captures both LBB and RBB to completely correct RBBB. **(C5,C6)** When conduction delay occurs at the terminal branch of the RBB, neither the unipolar nor bipolar LBBP can bypass the blockage. **(D)** A case with no significant changes in QRS morphology and duration of RBBB under different LBBP modes, suggesting that RBB distal block might be involved.

Although the excitation sequence of LBBP in RBBB patients is comparable to that of the original, the morphology of paced RBBB differs dramatically from that of intrinsic RBBB. Gao et al. (19) compared the ECG characteristics of LBBP to those of intrinsic RBBB, and discovered that the majority of the QRS morphology in lead V1 of LBBP showed a Qr pattern, whereas the majority of intrinsic RBBB showed a rsR’ pattern. Furthermore, they described an RBBB patient who completed SLBBP with the same terminal R’ wave duration of 76 ms as the intrinsic, but which reduced to 58 ms as output increased, suggesting that NSLBBP could compensate for RV delay by capturing a portion of cardiomyocytes, which is consistent with the findings of the other studies (22, 23). According to Li et al. (15), 8 atrioventricular block (AVB) patients with RBBB completed LBBP, with five RBBB corrected successfully by bipolar LBBP at a low output or unipolar pacing at relatively high output. Several other studies have also suggested that LBBP might be a viable choice to correct the RBBB (24, 25). Lin et al. (26) employed unipolar LBBP to shorten the RBBB duration from 137.7 ± 19.2 ms to 118.7 ± 6.7 ms, and bipolar LBBP to shorten the RBBB duration even further to 105.0 ± 5.0 ms, eliminating interventricular conduction delay. Previous studies have also shown that LBBP can shorten the QRS duration of intrinsic RBBB by about 30ms (27), as summarized in Table 1. In the following article, we present two cases of LBBP shortening RBBB and not shortening RBBB (Figures 1A,D), and the sheath angiography of the first case (Figure 1B). We will focus on the potential mechanisms underlying paced QRS narrowing during LBBP in RBBB patients (Figure 1C), including the anatomy and electrophysiology of the His-Purkinje system, classical and possible alternative understandings of longitudinal dissociation and transverse interconnection, output dependence of transition from SLBBP to NSLBBP, components captured of unipolar tip pacing configuration (UTP) and bipolar tip pacing configuration (BTP).

**TABLE 1:** Summary of studies demonstrating paced QRS narrowing during LBBP in RBBB.

Study	RBBB QRS duration (ms)	Paced QRS duration (ms)	Stim-LVAT (ms)	Number of RBBB patients	Success narrowing rate
Li et al. (15)	120	106	None	8	62.5%
Zhu et al. (22)	169	114 (low output UTP)/ 104 (high output UTP)	80	1	None
Li et al. (23)	143.1 ± 16.6	122.9 ± 10.3	77.4 ± 8.0 (low output UTP)/ 75.8 ± 7.5 (high output UTP)	27	88.8%
Jiang et al. (24)	150 ± 13	121 ± 15	86 ± 15	33	75.7%
Chu et al. (25)	141	109 (low output UTP)/ 106 (high output UTP)	None	1	None
Lin et al. (26)	137.7 ± 19.2	118.7 ± 6.7 (UTP)/105.0 ± 5.0 (BTP)	82.0 ± 5.2 (UTP)/ 78.3 ± 3.9 (BTP)	6	75% (BTP)
Zhu et al. (27)	144.31 ± 4.83	114.26 ± 5.09 (UTP)/ 116.7 ± 46.29 (BTP)	None	32	None

*LBBP, left bundle branch pacing; RBBB, right bundle branch block; Stim-LVAT, stimulus to left ventricular peak activation time; UTP, unipolar tip pacing configuration; BTP, bipolar tip pacing configuration.*

## Anatomy and Electrophysiology of His-Purkinje System

The His-Purkinje system is composed predominantly of longitudinally oriented Purkinje cells, which have a conduction velocity of 2.3 m/s and are specialized for rapid conduction, whereas the ventricular muscle is composed of working myocardial cells with typical intercalated discs and has a conduction velocity is only 0.75 m/s (28, 29). His bundle travels within the inferior margin of the membranous septum before dividing into the LBB and right bundle branch (RBB) at the subjacent left side of the crest of the muscular interventricular septum (30).

The LBB is a broad ribbon-like structure that emerges beneath the endocardium of the non-coronary cusp of the aortic valve and divides into a thin left anterior fascicle (LAF) and a broad left posterior fascicle (LPF) (29, 31, 32). The ribbon-like structure and rich interfascicular connections of LBB provide an anatomical foundation for the LBBP. The RBB is a cord-like structure that most commonly originates at an obtuse angle from the His bundle (HB) or merges as a continuation of a rightward HB (29, 30, 33). Because of its slender anatomical structure and blood supply only from the right coronary artery, the RBB is prone to injury.

Longitudinal dissociation theory, that is, LBB and RBB were predominantly separated longitudinally inside the HB by collagen sheaths (29, 34), but only at a distance of less than 2-3 mm (35). The existence of transverse interconnection in the HB and the proximal bundle branches was proven by Lazzara et al. (36), and the existence of transverse interconnection in RBB was undisputed. RBBB narrowing by LBBP may be transversally propagated from LBB to RBB by stimulation bypassing the blocking site. However, the transverse velocity of the bundle branches is significantly lower than longitudinal velocity (36), it is not clear whether the lateral capture of RBB can compensate for the excitation delay of RV caused by intrinsic RBBB. Besides, if transverse interconnection coexists alongside longitudinal dissociation, LBBP should not take an RBBB pattern when the right conduction system is normal, yet this is not the case.

## Output Dependence of Transition From SLBBP to NSLBBP

SLBBP is characterized by the isoelectric interval between the pacing artifact and the V wave in the intracardiac electrocardiogram, which indicates that only left conduction system is captured, resulting a typical RBBB pattern (17, 21). In patients with complete RBBB, the terminal R’ duration in lead V1 after SLBBP was consistent with the original, indicating that SLBBP could only accelerate LV excitation (19). SLBBP transforms into NSLBBP as output increases, capturing LBB and surrounding myocardium, presenting an atypical RBBB pattern, and shortening QRS duration (17). The terminal R’ duration of intrinsic RBBB in lead V1 similarly decreased as the output increased (19). However, the stimulus to left ventricular peak activation time (Stim-LVAT) remains constant and short in both SLBBP and NSLBBP regardless of output.

Li et al. (23) believed that low-output LBBP might capture LBB and surrounding myocardium, resulting in an incomplete RBBB pattern due to the delayed conduction of excitation to the distal RBB. High-output stimulation, on the other hand, was able to capture LBB, surrounding myocardium and RBB, shortening the RBBB QRS duration even further (15) (Figures 1A,C1–C3). While the emphasis remained on longitudinal dissociation theory and the necessity for high pacing output to overcome the resistivity of the fibrous sheath encasing the RBB within the HB. Finally, it is possible that, when the output increases, LBBP can capture more myocardium around LBB and partially compensate for the RV excitation delay caused by RBBB, thereby shortening the paced QRS duration, and even capturing RBB beyond the conduction block to completely correct RBBB.

## Unipolar Tip Pacing and Bipolar Tip Pacing Configuration

The paced QRS complex with BTP is differs from that with UTP, probably due to anodal capture during bipolar pacing (37). RV anodal capture is observed during cardiac resynchronization therapy treatment using an LV tip-RV ring pacing mode. The high output RV ring anodal capture necessary to stimulate the myocardium might induce depolarization and hyperpolarization regions around the RV ring, that is, virtual electrode polarization effect (38), which can improve cardiac contractility and accelerate conduction velocity (39).

Shimeno et al. (18) observed that the mean threshold of anodal capture in NSLBBP was 4.9 ± 1.2V @ 0.4ms, and that the paced QRS duration was significantly shorter than that without anodal capture (121 ± 9 ms vs. 135 ± 8 ms). Similarly, the average threshold for simultaneous capture of LBB and RBB in cathode tip and anode ring bipolar pacing mode in Lin et al. ’s study was 2.7 V @ 0.5 ms (26). LBBP with BTP configuration might also narrow the intrinsic RBBB duration (15, 23). This could be due to anodal capture, in which the anode ring penetrated the right side of the septum in a BTP configuration, allowing LBBP to stimulate the left and right septal myocardium as well as left conduction system simultaneously, partially compensating for the RV excitation delay caused by intrinsic RBBB (Figures 1A,C4). However, the output required to correct RBBB by anodal capture has not been reported, necessitating further study.

## The Blockage Site of the RBB and the Lead Tip Site of the LBBP

The location of RBBB is quite crucial. The existence of two types of RBBB has been confirmed: proximal RBBB, in which conduction interruption occurs at the main right branch of HB, and distal RBBB, in which conduction delay occurs at the terminal part of the RBB (40). According to the longitudinal dissociation theory, a high percentage of RBBB may be in the main right branch of HB (29, 34). However, pacing at the LBB trunk, LAF, and LPF resulted in similar intraventricular and interventricular electrical synchrony, suggesting that the lead tip site of LBBP may not be so important (41). The blockade point in proximal RBBB is above the pacing site of LBBP. Stimulation of LBBP can bypass the blockage to capture RBB by the transverse interconnection between LBB and RBB or anodal capture of anode ring. However, it may be difficult for LBBP to capture RBB in the case of distal RBBB (Figures 1C5,C6,D), since it has been observed that RBB was injured at anatomic bifurcation could not be corrected by LBBP regardless of the pacing output (23, 42).

## Discussion

The precise mechanisms underlying the paced QRS narrowing during LBBP in RBBB patients remain unclear and are likely multifactorial. The possible mechanism is that high output unipolar pacing overcomes the resistivity of fibrous sheaths of longitudinal dissociation and captures RBB by bypassing the blockage through transverse interconnection, or excites a part of the right septal myocardium by anodal capture of bipolar pacing to compensate for RV delay under the prerequisite that pacing lead of LBBP is placed beyond the block site of RBBB.

RBBB is the electrocardiographic reflection of RV excitation delay caused by RBB sclerosis, fibrosis, or necrosis, and it is associated with an elevated risk of all-cause mortality in patients with cardiac and pulmonary disease. The intrinsic RBBB may be corrected with traditional RVP by adjusting the atrioventricular interval to achieve optimal fusion with the intact LBB, maintaining the physiological LV excitation while correcting the delayed RV excitation. However, many RBBB patients who require a pacemaker may develop complete AVB that is unable to achieve fusion and necessitates 100% RVP. Furthermore, during exercise, atrioventricular conduction may shorten, reducing optimal fusion to some extent. HBP has been reported as a viable alternative for cardiac resynchronization therapy in RBBB patients with advanced heart failure, reduced LV ejection fraction and wide QRS duration (7–9). However, its development has been limited by unsatisfactory electrical parameters and a low success rate. LBBP directly captures LBB to accelerate left ventricular lateral wall excitation to achieve LV synchronization similar to HBP, but to increase interventricular dyssynchronization to result in “iatrogenic” RBBB (16, 20), the long-term outcome of this accompanying effect is unclear. In some pacing configurations, such as high output UTP or BTP, LBBP can partially or even completely correct intrinsic RBBB. In addition, the combination of LBBP and RVP can to achieve interventricular synchronization with proper biventricular pacing interval. In RBBB patients with high pacing percentage is expected, such as high-degree AVB, more physiological LBBP should be considered. This has sparked interest in whether LBBP can eliminate accompanying RBBB, and even whether LBBP can be an effective pacing therapy for RBBB patients with pacing indications to achieve more homogenous and physiologic interventricular synchronization rather than only intraventricular synchronization. It also makes sense to optimize the structure of pacing leads, such as adjusting the interelectrode distance so that the anode ring may be implanted more readily into the RBB region.

## Author Contributions

KZ: conceptualization and writing—original draft preparation. YS: writing—original draft preparation. ML, YD, LL, GL, JL, and XW: contribute to our revised draft and provide useful comments. DC and QL: supervision and writing—reviewing and editing. All authors contributed to the article and approved the submitted version.

## Conflict of Interest

The authors declare that the research was conducted in the absence of any commercial or financial relationships that could be construed as a potential conflict of interest.

## Publisher’s Note

All claims expressed in this article are solely those of the authors and do not necessarily represent those of their affiliated organizations, or those of the publisher, the editors and the reviewers. Any product that may be evaluated in this article, or claim that may be made by its manufacturer, is not guaranteed or endorsed by the publisher.
